# Role of the PE/PPE Family in Host–Pathogen Interactions and Prospects for Anti-Tuberculosis Vaccine and Diagnostic Tool Design

**DOI:** 10.3389/fcimb.2020.594288

**Published:** 2020-11-26

**Authors:** Jianing Qian, Run Chen, Honghai Wang, Xuelian Zhang

**Affiliations:** ^1^State Key Laboratory of Genetic Engineering, School of Life Science, Fudan University, Shanghai, China; ^2^Shanghai Engineering Research Center of Industrial Microorganisms, Fudan University, Shanghai, China

**Keywords:** PE/PPE proteins, *Mycobacterium tuberculosis*, macrophages, host–pathogen interaction, vaccine

## Abstract

The *pe/ppe* genes are found in pathogenic, slow-growing *Mycobacterium tuberculosis* and other *M. tuberculosis* complex (MTBC) species. These genes are considered key factors in host-pathogen interactions. Although the function of most PE/PPE family proteins remains unclear, accumulating evidence suggests that this family is involved in *M. tuberculosis* infection. Here, we review the role of PE/PPE proteins, which are believed to be linked to the ESX system function. Further, we highlight the reported functions of PE/PPE proteins, including their roles in host cell interaction, immune response regulation, and cell fate determination during complex host-pathogen processes. Finally, we propose future directions for PE/PPE protein research and consider how the current knowledge might be applied to design more specific diagnostics and effective vaccines for global tuberculosis control.

## Introduction

*Mycobacterium tuberculosis* (Mtb), the causative pathogen of tuberculosis (TB), is an extremely successful intracellular pathogen. The interactions between Mtb and host immune system determine the outcome of Mtb infection. PE/PPE families are seemingly related to mycobacteria pathogenicity, as its members are abundant in pathogenic mycobacteria (Akhter et al., 2012; Li et al., 2019) and less present in nonpathogenic mycobacteria (McGuire et al., 2012). Previous reviews have discussed the *pe/ppe* genes evolution (Fishbein et al., 2015), the expression and regulatory role of PE/PPE proteins (Li et al., 2019), as well as the relation with virulence and host cell fate (Yu et al., 2019).

Given the importance of the PE/PPE family in host-pathogen interactions, herein, we summarize the latest experimental advances in PE/PPE protein interactions with host cells and provide a comprehensive overview of the involvement in macrophage processing of Mtb, such as adhesion, receptor interactions, immune response, environmental stress resistance, phagocytosis, intracellular survival, and cell fate regulation. This information may contribute to tuberculosis future intervention strategies, such as improved diagnostic tools and vaccine candidates.

## Functional Relationships Between Pe/Ppe Proteins and The Esx Secretion System

Currently, the original evolution of the *pe/ppe* gene families remains unclear. However, the ancestral *pe/ppe* gene family is reportedly related to the ESX (early secretory antigen target 6 system) protein family (Gey van Pittius et al., 2006). The *pe/ppe* genes seem to have evolved and duplicated in association with the duplication of the five *esx* gene cluster regions in the Mtb genome (Gey van Pittius et al., 2006; Akhter et al., 2012; Fishbein et al., 2015) that can be inferred from the most primitive ESX-4, which has no PE/PPE proteins among its components, in contrast to the more recent ESX-5, which has two PE (PE18 and PE19) and three PPE (PPE25, PPE26, and PPE27) proteins (Majlessi et al., 2015). Besides, the recently evolved PE_PGRS (polymorphic GC-rich sequences) and PPE_MPTR (major polymorphic tandem repeats) subfamilies are believed to have originated from *pe/ppe* genes within the ESX-5 cluster (Gey van Pittius et al., 2006).

Recent studies have indicated that the ESX system contributes to PE/PPE protein export, and, likewise, ESX system protein secretion is related to that of PE/PPE proteins. Genes in ESX-1 locus that encode secreted proteins EsxA and EsxB are flanked directly upstream by *pe35* and *ppe68* (Majlessi et al., 2015). PPE68 and PE35 are required for Mtb virulence (Sassetti and Rubin, 2003; Jiang et al., 2016), and PE35 is required for EsxA and EsxB secretion (Brodin et al., 2006; Chen et al., 2013).

ESX−5 is related to the virulence of pathogenic mycobacteria. Deletion of *ppe25*, *pe18*, *ppe26*, *ppe27*, and *pe19* significantly attenuated the virulence in mouse models. Further, ESX-5 inactivation Mtb and *Mycobacterium marinum* mutants fail to secrete several PE/PPE proteins, many of which are not encoded by the *esx-5* locus, suggestive of a loss of the ability to transport PE/PPE proteins across mycobacterial cell envelope (Bottai et al., 2012). In addition, the expression of PE19 enhances envelope permeability inducing higher pathogenic sensitivity (Ramakrishnan et al., 2016). These results strongly suggest that PE/PPE proteins of ESX-5 locus are required for ESX-5 mediated protein export.

Although, to a lesser extent than ESX-1 and ESX-5, the correlation between ESX-3 and PPE-related functions has also been studied. Products of the *esx-3* gene locus, which contains *pe5* and *ppe4*, carry out the essential function of iron/zinc acquisition (Serafini et al., 2009; Siegrist et al., 2009). PE5 forms a heterodimer with PE4 to utilize iron from the intracellular host space (Tufariello et al., 2016). In contrast to other members, the exact function of *esx-2*, including *pe36* and *ppe69*, remains undefined.

Overall, due to the difficulty of recovering stable soluble recombinant PE/PPE proteins, knowledge of their biophysical structure remains insufficient to clarify the secretory interaction between PE/PPE proteins and the ESX system. However, it is worth mentioning that the ESX secretion-associated protein G (EspG), the homolog of the ESX system, recognizes its cognate PE/PPE protein, maintaining it in a stable conformation and promoting secretion (Daleke et al., 2012). The crystal structure of the PE25-PPE41-EspG_5_ complex yielded valuable information regarding the cross-talk between EspGs and different PE/PPE proteins (Ekiert and Cox, 2014; Korotkova et al., 2014).

## Role of PE/PPE Proteins in Host–Pathogen Interactions

**Figure 1:**
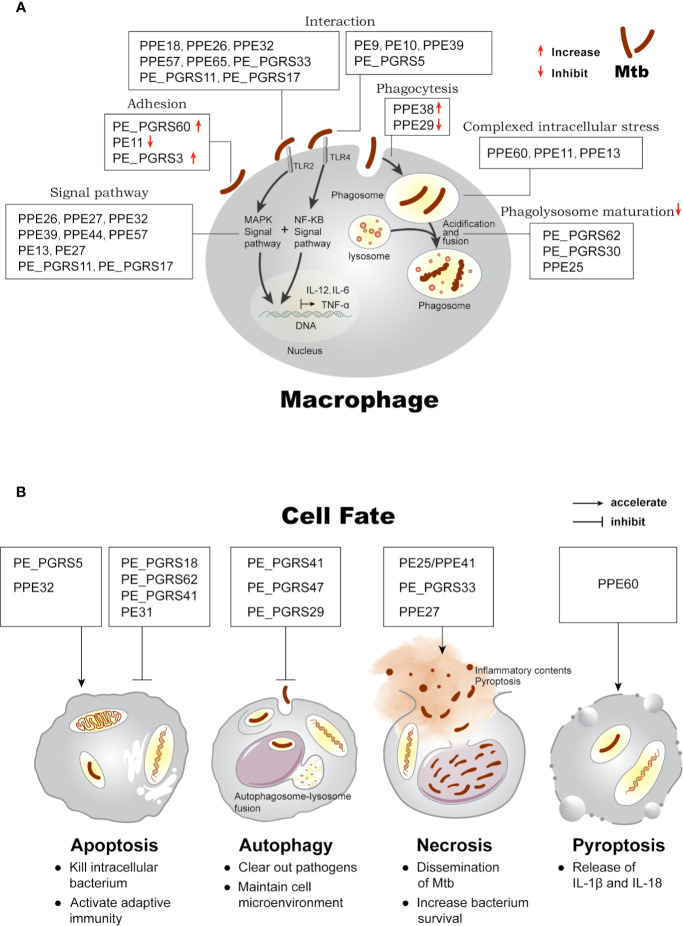
Effects of PE/PPE proteins on the interaction between Mtb and macrophage. **(A)** PE/PPE proteins in each frame are shown to affect each stage of host-pathogen interaction, including in cell adhesion and binding to TLR2 or TLR4 receptors, mediating downstream immune signal pathway, inhibiting or increasing phagocytosis of the bacilli, surviving under intracellular stress, and inhibiting phagolysosome maturation in macrophage. **(B)** PE/PPE proteins regulate four major outcomes observed in macrophage after Mtb infection.

Based on the different stages of interaction with the host, we summarize the function and localization of PE/PPE proteins in **Supplementary Table 1** and highlight the intriguing roles in **Figure 1A**: (i) mediating immune responses through cell surface adhesion or receptor binding; (ii) surviving under intracellular stress, phagocytosis, and phagolysosome maturation; (iii) determination of cell fate.

### Roles of PPE Proteins in Interactions With Host Cells and Immune Regulation

Surface exposure or secretion into the extracellular environment allows PE/PPE proteins to interact with their host targets directly. Some proteins reportedly interact with receptors like TLR2/4 on the surface of macrophages, activating downstream signaling pathways. The PE9-PE10 complex (Tiwari et al., 2015), PPE39 (Choi et al., 2019), and PE_PGRS5 (Grover et al., 2018) interact with TLR4 to activate downstream signaling and modulate cytokine production. Furthermore, various PE/PPE proteins can interact with TLR2, including PPE26 (Su et al., 2015), PPE32 (Deng et al., 2014), PPE57 (Xu et al., 2015), PPE65 (Qureshi et al., 2019), PE_PGRS33 (Basu et al., 2007; Zumbo et al., 2013; Palucci et al., 2016), and PE_PGRS11 (Bansal et al., 2010). PPE18 can stimulate IL-10 secretion, which might induce Th2 type response *via* interacting with TLR2 (Nair et al., 2009), and further was defined to inhibit the production of NF-κB/rel-mediated pro-inflammatory cytokine by upregulating suppressor of cytokine signaling 3 protein (SOCS3) (Nair et al., 2011). Besides, PE_PGRS17 was found to mature DCs *via* TLR2 (Bansal et al., 2010) and cause host cell death and cytokine secretion *via* Erk kinase, eventually enhancing intracellular survival (Chen et al., 2013).

Generally, the binding of PE/PPE proteins to cell surface receptors activates downstream signaling pathways, including NF-κB and MAPK (p38, JNK, and ERK), which affect cytokine production, leading to a pro-inflammatory or anti-inflammatory response. PPE27 overexpressed strain showed a strengthened ability to induce nitric oxide (NO) and inhibiting IL-6 production, which was abolished by NF-κB, p38, and ERK inhibitors (Yang G et al., 2017). PPE39, a PE/PPE protein defined in hypervirulent strain Beijing/K, exhibited its ability to mature DCs and activate Th1 immune response through NF-κB and MAPK, which functioned as TLR4 agonist (Choi et al., 2019). A series of proteins, including PE13 (Li et al., 2016), PE27 (Kim et al., 2016), PPE26 (Su et al., 2015), PPE32 (Deng et al., 2014), PPE44 (Yu et al., 2017), PPE57 (Xu et al., 2015), PE_PGRS11, and PE_PGRS17 (Bansal et al., 2010) similarly regulate the cytokine profile *via* NF-κB and MAPK signaling.

PE/PPE protein effects on mycobacterial invasion and macrophage phagocytosis have also been suggested. PPE38-mutant of *Mycobacterium marinum* exhibited significantly higher invasion efficiency (Dong et al., 2012), while the phagocytosis ratio of PPE29 mutants was expectedly reduced (Meng et al., 2017).

Adherence to the cell surface is another prerequisite for bacterial invasion. Recent reports revealed that PE11 knockdown strains could significantly enhance fibronectin attachment protein production, contributing to the attachment to the host extracellular matrix (Rastogi et al., 2017). PE_PGRS60 can bind to fibronectin, which results in enhanced adhesion and invasion (Meena and Meena, 2016).

### Roles of PE/PPE Proteins in Intracellular Survival

Upon entry into macrophages, pathogens adapt to the intracellular environment, such as low pH, reactive oxygen, and nitrogen species, thus creating its own niche. Besides, PPE60 (Gong et al., 2019) and PE13 (Li et al., 2016) can enhance cell resistance to low pH, surface stresses, and antibiotic exposure to increase intracellular survival. PPE11 has also increased early bacterial survival rate under conditions similar to the intracellular macrophage environment, such as the presence of lysozymes, acidic, and active nitrogen intermediates (RNI), and maintains a high bacterial load in mouse tissue, worsening organ pathology (Peng et al., 2018).

Once adapted to the harsh conditions, Mtb survives in macrophages by preventing phagosomal acidification and phagosome-lysosome fusion. PE_PGRS30 and PE_PGRS47 knockout strains lost the ability to inhibit phagosome fusion (Iantomasi et al., 2012; Saini et al., 2016). Similarly, overexpressed PE_PGRS62 significantly inhibits phagosome maturation (Huang et al., 2012; Thi et al., 2013; Long et al., 2019). During phagocytosis, the transcription level of PPE25 is upregulated, and PPE25 mutant strain loses its ability to replicate within macrophages and prevent phagosome-lysosome fusion (Jha et al., 2010).

### PE/PPE Proteins Are Involved in the Determination of Cell Fate

Further, PE/PPE proteins are believed to have roles in host defense mechanism which limit Mtb survival or are closely associated with the intracellular persistence and proliferation, eventually inducing host cells three major outcomes as shown in **Figure 1B**: (i) apoptosis, a form of programmed cell death that is proactively regulated by host cells (Fink and Cookson, 2005); (ii) autophagy, a host degradation system that can resolve infection (Mariño et al., 2014); and (iii) necrosis, a form of passive cell death triggered by external stimuli (Fink and Cookson, 2005).

Cell apoptosis can affect intracellular bacterial viability (Duan et al., 2002). Recent studies indicate that PPE32 (Deng et al., 2016) and PE_PGRS5 (Grover et al., 2018) are involved in ER stress-mediated cell apoptosis. Conversely, PE_PGRS62 (Long et al., 2019) and PE_PGRS18 (Yang W. et al., 2017) can decrease apoptosis and enhance survival rate. PE31 increased guanylate-binding protein-1 (GBP-1) expression and inhibited caspase-3 activation and macrophage apoptosis through the NF-κB pathway (Ali et al., 2020). Although apoptosis caused by some bacterial proteins favors bacterial survival, it also helps to kill intracellular bacteria and activate adaptive immunity (Schaible et al., 2003; Srinivasan et al., 2014). PE/PPE proteins with pro-apoptotic activity might serve as candidates for vaccine development.

Autophagy is related to autolysosome formation, which helps host cell clear out the pathogen, but an aberrant autolysosome may consume most cellular proteins and organelles, thus inducing autophagic cell death (Mariño et al., 2014). PE_ PGRS41 (Deng et al., 2017) and PE_PGRS47 (Saini et al., 2016) have been proved to inhibit autophagy from allowing pathogen survival. A recent report revealed that ubiquitinated PE_PGRS29 could recruit autophagy receptor p62 and deliver Mtb into autophagosomes. Disruption of the interaction between PE_PGRS29 and ubiquitin attenuates Mtb xenophagic clearance, leading to an enhanced bacterial load and an elevated inflammatory response (Chai et al., 2019).

Cell necrosis is involved in the dissemination and virulence of Mtb because it results in the release and spread of tuberculosis-causing pathogens (Behar et al., 2010). Such a function has been reported for PE25-PPE41 complex (Tundup et al., 2014), PE_PGRS33 (Dheenadhayalan et al., 2006), and PPE27 (Yang G. et al., 2017).

In addition, a PPE60-overexpressing strain has recently been found to increase intracebllular survival and shift cell fate to pyroptosis, a newly defined form of programmed cell death, which is correlated with restriction of intracellular growth and enhanced host immune response (Gong et al., 2019; Chai et al., 2020), and with the maturation of IL-1β and IL-18 (Beckwith et al., 2020).

## Future Applications of Pe/Ppe Family Proteins In Tb Vaccine Design and Diagnostic Tool Development

Serological antibody assays are routinely performed; however, there is no gold standard in TB serological diagnosis. PE35, an RD1-encoded antigen, can significantly discriminate pulmonary or extra-pulmonary TB patients with healthy BCG-vaccinated individuals (Mukherjee et al., 2007). Another good example is PPE17, whose N-terminal induces high immunogenic response and had greater potential to be a sero-diagnostic marker than full-length PPE17 (Abraham et al., 2017), which could screen the latently infected subjects (Abraham et al., 2018). PPE2 may also serve as a serodiagnosis marker to detect the extra-pulmonary and smear-negative pulmonary cases (Abraham et al., 2014).

The highly immunogenic properties of PE/PPE proteins have been demonstrated by the investigation of IFN-γ T cell responses generated during infection. CD4^+^-specific epitope-rich PE/PPE proteins, including PE18, PE19, PPE25, PPE26, and PPE27, are potent inducers of cell-mediated immune responses (Sayes et al., 2012). Vordermeier *et al*. examined cellular immune responses against a panel of 36 PE/PPE proteins during human and bovine infection and observed that many were major targets of the cellular immune response to tuberculosis. The specific HLA-A*0201-restricted epitopes of PPE68 also elicit a potent cellular response (Duan et al., 2015). Additionally, the PE5 protein and EsxI have been proven as a diagnostic antigen of bovine tuberculosis during intradermal tests (Melo et al., 2015). A combination of PPE57 can also increase the sensitivity of ESAT-6 or CFP-10 in the IFN-γ releasing assay for detecting active TB (Chen et al., 2009). The highly cellular immune response indicates that PE/PPE proteins may be better diagnostic and vaccine candidates (Vordermeier et al., 2012).

Numerous studies have also been carried out to assess the potential of PE/PPE proteins as candidate vaccine antigens. Several attempts seem promising. In dendritic cells, which serve as the most efficient antigen-presenting cells, PE27 (Kim et al., 2016), PPE39 (Choi et al., 2019), and PPE60 (Su et al., 2018) could change the cytokine profile toward a pro-inflammatory immune response, suggesting the possibility to be subunit vaccines for tuberculosis. In macrophages, PPE57 (Xu et al., 2015), PPE26 (Su et al., 2015), and PE3 (Singh et al., 2013) were also found to generate a protective immune response. Further, PPE44, HspX, and EsxV could enhance BCG protective efficacy (Mansury et al., 2019). Another vaccine candidate worth mentioning is the attenuated MtbΔ*ppe25-pe19* strain, which outcompeted BCG protective capacity (Sayes et al., 2012). Notably, the contribution of PE-specific and PPE-specific T helper cell 1 (Th1) effector cells in protective immunity against mycobacteria has been recently identified (Sayes et al., 2016).

However, there is a downside to the use of PE/PPE proteins in vaccines, as many of them are believed to hamper the host inflammatory response to evade immune surveillance, thus supporting the development of an immunopathological response. PE32/PPE65 (Khubaib et al., 2016), PPE37 (Daim et al., 2011), and PE25/PPE14 (Chen et al., 2015) were found to tilt the Th1 response toward a Th2 response, which favors the intracellular survival of bacteria. In addition, PE/PPE proteins are polymorphic within clinical isolates (Hebert et al., 2007) and can be degradation-resistant, limiting MHC processing (Koh et al., 2009). However, researchers surprisingly found that the PPE18 protein, which upregulated IL-10 production (Nair et al., 2009) and inhibited the inflammatory response, could be explored as a therapeutic for sepsis caused by exaggerated inflammatory responses (Ahmed et al., 2018). Thorough characterization of candidates or exclusive use of the immunodominant epitopes of PE/PPE proteins may facilitate vaccine development.

## Discussion

Since its discovery over 20 years ago, PE/PPE family has been recognized as exclusive to mycobacteria, especially in pathogenic species. Several studies have defined that PE/PPE protein expression is linked to ESX gene clusters is now well-established (Bottai et al., 2012; Sayes et al., 2012). Improved knowledge of the ESX system function has dramatically advanced our understanding of the biological function of specific PE/PPE proteins. Moreover, structural biology studies have started to solve and explain the roles of protein complexes involved in PE-PPE and ESX secretion (Ekiert and Cox, 2014). However, the biology and structure of PE/PPE proteins remain far less understood than other mycobacterial proteins. Elucidating the structure of PE/PPE proteins and their complexes with ESX systems will be pivotal to a more comprehensive mechanistic understanding of how the PE/PPE protein family, in association with the ESX secretion system, contributes to the pathogenicity of Mtb. This is of importance for obtaining further insights into the virulence strategies of mycobacteria, and may provide novel targets for antimycobacterial treatment.

Another feature of the PE/PPE proteins is that they are often found as co-operonic pairs of mostly one PE- and one PPE-coding gene, whose products interact with each other (Akhter et al., 2012) and are believed to assemble as heterodimers (Strong et al., 2006; Tundup et al., 2006; Tiwari et al., 2014). Such interactions have been predicted using bioinformatic tools (Riley et al., 2008) and proven through experimental evidence, as in the cases of PPE41 and PE25 (Tundup et al., 2006), PE35 and PPE68 (Tiwari et al., 2014), as well as PE19 and PPE51 (Wang et al., 2020). Korycka-Machała *et al*. found that PPE51 deletion rendered Mtb cells unable to replicate in propionamide, glucose, or glycerol. Further, some PE/PPE proteins, such as PE20/PPE31 and PE32/PPE65, are required by Mtb during Mg^2+^ and PO3^2−^ restriction (Wang et al., 2020). PPE36/PPE62 (Mitra et al., 2019) and PPE37 (Tullius et al., 2018) are essential for heme-iron acquisition and Mtb growth. Additionally, mutant PPE51 and PE19 strains developed resistance to 3bMP1, a compound with anti-tuberculosis activity (Wang et al., 2020). These data suggest that at least some PE/PPE proteins appear to act as solute-selective pores, allowing the access of exogenous agents or nutrients required for proliferation. Thus, focusing on genetic mutations of *pe*/*ppe* family members, which are often eliminated when analyzing next-generation sequencing data of clinically drug-resistant strains, may help discover anti-tuberculosis drug resistance mechanisms. In summary, we believe that the PE/PPE family will remain a highly active area of research with various exciting features yet to be discovered.

## Author Contributions

JQ wrote the manuscript draft. XZ supervised and revised the manuscript. RC and HW gave some suggestions. All authors contributed to the article and approved the submitted version. Key scientific and technological projects of Xinjiang production and Construction Corps (2020AB015).

## Funding

This work was supported by the National Natural Science Foundation of China (81673482 and 81971898), SKLGE-1912 and Key scientific and technological projects of Xinjiang production and Construction Corps (2020AB015).

## Conflict of Interest

The authors declare that the research was conducted in the absence of any commercial or financial relationships that could be construed as a potential conflict of interest.
