# Supplementary material for: Role of the PE/PPE Family in Host–Pathogen Interactions and Prospects for Anti-Tuberculosis Vaccine and Diagnostic Tool Design
Source: Front Cell Infect Microbiol. 2020 Nov 26;10:594288. doi: 10.3389/fcimb.2020.594288 (PMC7726347; doi:10.3389/fcimb.2020.594288)
Supplement: Supplementary file 1 [file Table_1.docx]

Supplementary Material

**Supplementary Table 1.** Summary of function, localization and operon-sharing protein of PE/PPE proteins from experimental data. (MET: Metabolism, IS: Intracellular survival, IR: Immune response, CA: Cell activity, CW: Cell wall)

| Rv number | PE/PPE name | Operon pairs | | Function | Localization | Reference |
| --- | --- | --- | --- | --- | --- | --- |
| Rv0151c | PE1 |  | MET | |  | (Divya M et al., 2018) |
| Rv0152c | PE2 |  | MET | |  | (Divya M et al., 2018) |
| Rv0159c | PE3 |  | IS, IR | |  | (Singh et al., 2013) |
| Rv0160c | PE4 |  | IS, IR | |  | (Singh et al., 2012) |
| Rv0256c | PPE2 |  | IS | | secretion | (Bhat et al., 2013, 2017) |
| Rv0278c | PE_PGRS3 |  | CA, IS, IR | | surface | (De Maio et al., 2018) |
| Rv0280 | PPE3 |  | IS | |  | (Meng et al., 2017) |
| Rv0285 | PE5 | PPE4 | MET (1), IS (2), IR (2) | | Surface (2) | (Tufariello et al., 2016) (1); (Tiwari et al., 2012) (2) |
| Rv0286 | PPE4 | PE5 | MET | |  | (Tufariello et al., 2016) |
| Rv0297 | PE_PGRS5 |  | CA, IS | |  | (Grover et al., 2018) |
| Rv0354c | PPE7 |  | CA (cell invasion) (1), IS (2), IR (2) | | surface | (Díaz et al., 2017) (1); (Meng et al., 2017) (2) |
| Rv0442c | PPE10 |  | capsular integrity (1), IS (1) | | CW (2) | (Ates et al., 2016) (1); (Tiwari et al., 2015)(2) |
| Rv0453 | PPE11 |  | CA, IS, IR | | CW | (Peng et al., 2018) |
| Rv0755c | PPE12 |  | IS, IR | |  | (Meng et al., 2017) |
| Rv0878c | PPE13 |  | IS | |  | (Meng et al., 2017) |
| Rv0915c | PPE14 | PE7 | IS | |  | (Meng et al., 2017) |
| Rv0977 | PE_PGRS16 |  | CA, IS, IR | | Surface | (Singh et al., 2008) |
| Rv0978c | PE_PGRS17 |  | CA, IS, IR | |  | (Chen et al., 2013) |
| Rv0980c | PE_PGRS18 |  | CA, IS, IR | | CW | (Yang et al., 2017b) |
| Rv1039c | PPE15 | PE8 | MET | |  | (Daniel et al., 2016) |
| Rv1088 | PE9 |  | CA (1), IR (2) | | CW (2) | (Díaz et al., 2016) (1); (Tiwari et al., 2015) (2) |
| Rv1089 | PE10 |  | CA, IR | | CW | (Tiwari et al., 2015) |
| Rv1168c | PPE17 | PE11 | IR (1) | | CW (2) | (Abraham et al., 2017) (1); (Donà et al., 2013)(2) |
| Rv1169c | lipX/PE11 | PPE17 | MET, IS, IR (1); CA (2) | | CW (1) | (Singh et al., 2016) (1); (Deng et al., 2015) (2) |
| Rv1196 | PPE18 | PE13 | CA, IR (1); IS (2) | |  | (Ahmed et al., 2018) (1); (Meng et al., 2017) (2) |
| Rv1361c | PPE19 |  | IR | |  | (Meng et al., 2017) |
| Rv1386 | PE15 | PPE20 | IS, IR | | surface | (Tiwari et al., 2012) |
| Rv1387 | PPE20 | PE15 | IS | |  | (Meng et al., 2017) |
| Rv1430 | PE16 |  | MET | |  | (Sultana et al., 2013) |
| Rv1441c | PE_PGRS26 |  | CA, IS, IR | | surface | (Singh et al., 2008) |
| Rv1646 | PE17 |  | CA, IS, IR | | CW | (Li et al., 2019) |
| Rv1651c | PE_PGRS30 |  | CA (1), IS (1), IR (2) | | CW (3) | (Iantomasi et al., 2012) (1); (Chatrath et al., 2016) (2); (Chatrath et al., 2014) (3) |
| Rv1753c | PPE24 |  | IS | |  | (Meng et al., 2017) |
| Rv1787 | PPE25 |  | CA, IS, IR | | CW | (Mi et al., 2017) |
| Rv1789 | PPE26 | PE18 | CA, IS, IR | | CW | (Mi et al., 2017) |
| Rv1790 | PPE27 |  | CA, IS, IR | | CW | (Yang et al., 2017a) |
| Rv1791 | PE19 |  | Cell wall permeability | | CW | (Ramakrishnan et al., 2016) |
| Rv1801 | PPE29 |  | CA | |  | (Meng et al., 2017) |
| Rv1807 | PPE31 | PE20 | IS | |  | (Meng et al., 2017) |
| Rv1808 | PPE32 |  | CA, IS, IR | | CW | (Deng et al., 2014, 2016) |
| Rv1818c | PE_PGRS33 |  | CA, IS, IR | | surface | (Singh et al., 2008; Zumbo et al., 2013) |
| Rv1917c | PPE34 |  | IR | |  | (Bansal et al., 2010) |
| Rv2108 | PPE36 | PE22 | IS (1) | | CW (2) | (Meng et al., 2017) (1); (Le Moigne et al., 2013)(2) |
| Rv2123 | PPE37 |  | MET (1), CA (2), IR (2) | |  | (Tullius et al., 2018) (1); (Ahmad et al., 2018) (2) |
| Rv2352c | PPE38 |  | CA, IR | | Surface | (Dong et al., 2012) |
| Rv2353c | PPE39 |  | IS | |  | (Choi et al., 2019) |
| Rv2396 | PE_PGRS41 |  | CA, IS, IR | | CW | (Deng et al., 2017) |
| Rv2430c | PPE41 | PE25 | CA, IR | |  | (Tundup et al., 2014) |
| Rv2431c | PE25 | PPE41 | CA, IR | |  | (Tundup et al., 2014) |
| Rv2741 | PE_PGRS47 |  | CA, IR | |  | (Saini et al., 2016) |
| Rv2769c | PE27 | PPE34 | CA, IR | |  | (Kim et al., 2016) |
| Rv2770c | PPE44 |  | CA, IS, IR | | CW | (Yu et al., 2017) |
| Rv3135 | PPE50 |  | IS | |  | (Meng et al., 2017) |
| Rv3136 | PPE51 | PE19 | IS (1), MET (2) | |  | Meng,2017(Meng et al., 2017) (1); (Korycka-Machała et al., 2020) (2) |
| Rv3347c | PPE55 |  | IR | |  | (Singh et al., 2005) |
| Rv3425 | PPE57 |  | CA, IR | | CW | (Xu et al., 2015) |
| Rv3477 | PE31 | PPE60 | CA, IS, IR | |  | (Ali et al., 2020) |
| Rv3478 | PPE60 | PE31 | CA, IS, IR | | CW | (Gong et al., 2019) |
| Rv3621c | PPE65 | PE32 | IR | |  | (Khubaib et al., 2016) |
| Rv3622c | PE32 | PPE65 | IR | |  | (Khubaib et al., 2016) |
| Rv3652 | PE_PGRS60 |  | fibronectin binding | |  | (Meena and Meena, 2016) |
| Rv3812 | PE_PGRS62 |  | CA (1), IS (1), IR (2) | | CW (1) | (Thi et al., 2013) (1); (Long et al., 2019)(2) |
| Rv3872 | PE35 | PPE68 | CA, IR | | CW | (Tiwari et al., 2014) |
| Rv3873 | PPE68 | PE35 | CA, IR (1), IS (2) | | CW (1) | (Tiwari et al., 2014)(1); (Meng et al., 2017) (2) |

Reference:

Abraham, P. R., Pathak, N., Pradhan, G., Sumanlatha, G., and Mukhopadhyay, S. (2017). The N-terminal domain of Mycobacterium tuberculosis PPE17 (Rv1168c) protein plays a dominant role in inducing antibody responses in active TB patients. *PLOS ONE* 12, e0179965. doi:10.1371/journal.pone.0179965.

Ahmad, J., Farhana, A., Pancsa, R., Arora, S. K., Srinivasan, A., Tyagi, A. K., et al. (2018). Contrasting Function of Structured N-Terminal and Unstructured C-Terminal Segments of Mycobacterium tuberculosis PPE37 Protein. *mBio* 9, e01712-17. doi:10.1128/mBio.01712-17.

Ahmed, A., Dolasia, K., and Mukhopadhyay, S. (2018). *Mycobacterium tuberculosis* PPE18 Protein Reduces Inflammation and Increases Survival in Animal Model of Sepsis. *J. Immunol.* 200, 3587–3598. doi:10.4049/jimmunol.1602065.

Ali, M. K., Zhen, G., Nzungize, L., Stojkoska, A., Duan, X., Li, C., et al. (2020). Mycobacterium tuberculosis PE31 (Rv3477) Attenuates Host Cell Apoptosis and Promotes Recombinant M. smegmatis Intracellular Survival via Up-regulating GTPase Guanylate Binding Protein-1. *Front. Cell. Infect. Microbiol.* 10, 40. doi:10.3389/fcimb.2020.00040.

Ates, L. S., van der Woude, A. D., Bestebroer, J., van Stempvoort, G., Musters, R. J. P., Garcia-Vallejo, J. J., et al. (2016). The ESX-5 System of Pathogenic Mycobacteria Is Involved In Capsule Integrity and Virulence through Its Substrate PPE10. *PLOS Pathog.* 12, e1005696. doi:10.1371/journal.ppat.1005696.

Bansal, K., Sinha, A. Y., Ghorpade, D. S., Togarsimalemath, S. K., Patil, S. A., Kaveri, S. V., et al. (2010). Src Homology 3-interacting Domain of Rv1917c of *Mycobacterium tuberculosis* Induces Selective Maturation of Human Dendritic Cells by Regulating PI3K-MAPK-NF-κB Signaling and Drives Th2 Immune Responses. *J. Biol. Chem.* 285, 36511–36522. doi:10.1074/jbc.M110.158055.

Bhat, K. H., Das, A., Srikantam, A., and Mukhopadhyay, S. (2013). PPE2 protein of *Mycobacterium tuberculosis* may inhibit nitric oxide in activated macrophages: *M. tuberculosis* PPE2 protein. *Ann. N. Y. Acad. Sci.* 1283, 97–101. doi:10.1111/nyas.12070.

Bhat, K. H., Srivastava, S., Kotturu, S. K., Ghosh, S., and Mukhopadhyay, S. (2017). The PPE2 protein of Mycobacterium tuberculosis translocates to host nucleus and inhibits nitric oxide production. *Sci. Rep.* 7, 39706. doi:10.1038/srep39706.

Chatrath, S., Gupta, V. K., Dixit, A., and Garg, L. C. (2016). PE_PGRS30 of Mycobacterium tuberculosis mediates suppression of proinflammatory immune response in macrophages through its PGRS and PE domains. *Microbes Infect.* 18, 536–542. doi:10.1016/j.micinf.2016.04.004.

Chatrath, S., Gupta, V. K., and Garg, L. C. (2014). The PGRS domain is responsible for translocation of PE_PGRS30 to cell poles while the PE and the C-terminal domains localize it to the cell wall. *FEBS Lett.* 588, 990–994. doi:10.1016/j.febslet.2014.01.059.

Chen, T., Zhao, Q., Li, W., and Xie, J. (2013). *Mycobacterium tuberculosis* PE_PGRS17 Promotes the Death of Host Cell and Cytokines Secretion via Erk Kinase Accompanying with Enhanced Survival of Recombinant *Mycobacterium smegmatis*. *J. Interferon Cytokine Res.* 33, 452–458. doi:10.1089/jir.2012.0083.

Choi, H.-H., Kwon, K. W., Han, S. J., Kang, S. M., Choi, E., Kim, A., et al. (2019). PPE39 of the *Mycobacterium tuberculosis* strain Beijing/K induces Th1-cell polarization through dendritic cell maturation. *J. Cell Sci.* 132, jcs228700. doi:10.1242/jcs.228700.

Daniel, J., Kapoor, N., Sirakova, T., Sinha, R., and Kolattukudy, P. (2016). The perilipin-like PPE15 protein in *Mycobacterium tuberculosis* is required for triacylglycerol accumulation under dormancy-inducing conditions: Mycobacterial PPE15 protein. *Mol. Microbiol.* 101, 784–794. doi:10.1111/mmi.13422.

De Maio, F., Battah, B., Palmieri, V., Petrone, L., Corrente, F., Salustri, A., et al. (2018). PE_PGRS3 of *Mycobacterium tuberculosis* is specifically expressed at low phosphate concentration, and its arginine-rich C-terminal domain mediates adhesion and persistence in host tissues when expressed in *Mycobacterium smegmatis*. *Cell. Microbiol.* 20, e12952. doi:10.1111/cmi.12952.

Deng, W., Li, W., Zeng, J., Zhao, Q., Li, C., Zhao, Y., et al. (2014). *Mycobacterium Tuberculosis* PPE Family Protein Rv1808 Manipulates Cytokines Profile via Co-Activation of MAPK and NF-κB Signaling Pathways. *Cell. Physiol. Biochem.* 33, 273–288. doi:10.1159/000356668.

Deng, W., Long, Q., Zeng, J., Li, P., Yang, W., Chen, X., et al. (2017). Mycobacterium tuberculosis PE_PGRS41 Enhances the Intracellular Survival of M. smegmatis within Macrophages Via Blocking Innate Immunity and Inhibition of Host Defense. *Sci. Rep.* 7, 46716. doi:10.1038/srep46716.

Deng, W., Yang, W., Zeng, J., Abdalla, A. E., and Xie, J. (2016). *Mycobacterium tuberculosis* PPE32 promotes cytokines production and host cell apoptosis through caspase cascade accompanying with enhanced ER stress response. *Oncotarget* 7. doi:10.18632/oncotarget.12030.

Deng, W., Zeng, J., Xiang, X., Li, P., and Xie, J. (2015). PE11 (Rv1169c) selectively alters fatty acid components of Mycobacterium smegmatis and host cell interleukin-6 level accompanied with cell death. *Front. Microbiol.* 6. doi:10.3389/fmicb.2015.00613.

Díaz, D. P., Ocampo, M., Pabón, L., Herrera, C., Patarroyo, M. A., Munoz, M., et al. (2016). Mycobacterium tuberculosis PE9 protein has high activity binding peptides which inhibit target cell invasion. *Int. J. Biol. Macromol.* 86, 646–655. doi:10.1016/j.ijbiomac.2015.12.081.

Díaz, D. P., Ocampo, M., Varela, Y., Curtidor, H., Patarroyo, M. A., and Patarroyo, M. E. (2017). Identifying and characterising PPE7 (Rv0354c) high activity binding peptides and their role in inhibiting cell invasion. *Mol. Cell. Biochem.* 430, 149–160. doi:10.1007/s11010-017-2962-8.

Divya M, B., Vemula, M., Balakrishnan, K., Banerjee, S., and Guruprasad, L. (2018). Mycobacterium tuberculosis PE1 and PE2 proteins carrying conserved α/β-serine hydrolase domain are esterases hydrolyzing short to medium chain p-nitrophenyl esters. *Prog. Biophys. Mol. Biol.* 140, 90–102. doi:10.1016/j.pbiomolbio.2018.04.012.

Donà, V., Ventura, M., Sali, M., Cascioferro, A., Provvedi, R., Palù, G., et al. (2013). The PPE Domain of PPE17 Is Responsible for Its Surface Localization and Can Be Used to Express Heterologous Proteins on the Mycobacterial Surface. *PLoS ONE* 8, e57517. doi:10.1371/journal.pone.0057517.

Dong, D., Wang, D., Li, M., Wang, H., Yu, J., Wang, C., et al. (2012). PPE38 Modulates the Innate Immune Response and Is Required for Mycobacterium marinum Virulence. *Infect. Immun.* 80, 43–54. doi:10.1128/IAI.05249-11.

Gong, Z., Kuang, Z., Li, H., Li, C., Ali, M. K., Huang, F., et al. (2019). Regulation of host cell pyroptosis and cytokines production by Mycobacterium tuberculosis effector PPE60 requires LUBAC mediated NF-κB signaling. *Cell. Immunol.* 335, 41–50. doi:10.1016/j.cellimm.2018.10.009.

Grover, S., Sharma, T., Singh, Y., Kohli, S., P., M., Singh, A., et al. (2018). The PGRS Domain of *Mycobacterium tuberculosis* PE_PGRS Protein Rv0297 Is Involved in Endoplasmic Reticulum Stress-Mediated Apoptosis through Toll-Like Receptor 4. *mBio* 9, e01017-18, /mbio/9/3/mBio.01017-18.atom. doi:10.1128/mBio.01017-18.

Iantomasi, R., Sali, M., Cascioferro, A., Palucci, I., Zumbo, A., Soldini, S., et al. (2012). PE_PGRS30 is required for the full virulence of Mycobacterium tuberculosis: PE_PGRS30 is an Mtb virulence factor. *Cell. Microbiol.* 14, 356–367. doi:10.1111/j.1462-5822.2011.01721.x.

Khubaib, M., Sheikh, J. A., Pandey, S., Srikanth, B., Bhuwan, M., Khan, N., et al. (2016). Mycobacterium tuberculosis Co-operonic PE32/PPE65 Proteins Alter Host Immune Responses by Hampering Th1 Response. *Front. Microbiol.* 7. doi:10.3389/fmicb.2016.00719.

Kim, W. S., Kim, J.-S., Cha, S. B., Kim, S. J., Kim, H., Kwon, K. W., et al. (2016). Mycobacterium tuberculosis PE27 activates dendritic cells and contributes to Th1-polarized memory immune responses during in vivo infection. *Immunobiology* 221, 440–453. doi:10.1016/j.imbio.2015.11.006.

Korycka-Machała, M., Pawełczyk, J., Borówka, P., Dziadek, B., Brzostek, A., Kawka, M., et al. (2020). PPE51 Is Involved in the Uptake of Disaccharides by Mycobacterium tuberculosis. *Cells* 9, 603. doi:10.3390/cells9030603.

Le Moigne, V., Le Moigne, D., and Mahana, W. (2013). Antibody response to Mycobacterium tuberculosis p27-PPE36 antigen in sera of pulmonary tuberculosis patients. *Tuberculosis* 93, 189–191. doi:10.1016/j.tube.2012.10.006.

Li, Z., Liu, H., Li, H., Dang, G., Cui, Z., Song, N., et al. (2019). PE17 protein from Mycobacterium tuberculosis enhances Mycobacterium smegmatis survival in macrophages and pathogenicity in mice. *Microb. Pathog.* 126, 63–73. doi:10.1016/j.micpath.2018.10.030.

Long, Q., Xiang, X., Yin, Q., Li, S., Yang, W., Sun, H., et al. (2019). PE_PGRS62 promotes the survival of *Mycobacterium smegmatis* within macrophages via disrupting ER stress‐mediated apoptosis. *J. Cell. Physiol.* 234, 19774–19784. doi:10.1002/jcp.28577.

Meena, L. S., and Meena, J. (2016). Cloning and characterization of a novel PE_PGRS60 protein (Rv3652) of *Mycobacterium tuberculosis* H _37_ Rv exhibit fibronectin-binding property: New FnBP from *M. tuberculosis*. *Biotechnol. Appl. Biochem.* 63, 525–531. doi:10.1002/bab.1411.

Meng, L., Tong, J., Wang, Q., Niu, C., and Gao, Q. (2017). Diverse effects of mycobacterial PPE proteins upon interaction with host macrophages. *FEMS Microbiol. Lett.*, fnx025. doi:10.1093/femsle/fnx025.

Mi, Y., Bao, L., Gu, D., Luo, T., Sun, C., and Yang, G. (2017). Mycobacterium tuberculosis PPE25 and PPE26 proteins expressed in Mycobacterium smegmatis modulate cytokine secretion in mouse macrophages and enhance mycobacterial survival. *Res. Microbiol.* 168, 234–243. doi:10.1016/j.resmic.2016.06.004.

Peng, X., Luo, T., Zhai, X., Zhang, C., Suo, J., Ma, P., et al. (2018). PPE11 of Mycobacterium tuberculosis can alter host inflammatory response and trigger cell death. *Microb. Pathog.* 126, 45–55. doi:10.1016/j.micpath.2018.10.031.

Ramakrishnan, P., Aagesen, A. M., McKinney, J. D., and Tischler, A. D. (2016). Mycobacterium tuberculosis Resists Stress by Regulating PE19 Expression. *Infect. Immun.* 84, 735–746. doi:10.1128/IAI.00942-15.

Saini, N. K., Baena, A., Ng, T. W., Venkataswamy, M. M., Kennedy, S. C., Kunnath-Velayudhan, S., et al. (2016). Suppression of autophagy and antigen presentation by Mycobacterium tuberculosis PE_PGRS47. *Nat. Microbiol.* 1, 16133. doi:10.1038/nmicrobiol.2016.133.

Singh, K. K., Dong, Y., Patibandla, S. A., McMurray, D. N., Arora, V. K., and Laal, S. (2005). Immunogenicity of the Mycobacterium tuberculosis PPE55 (Rv3347c) Protein during Incipient and Clinical Tuberculosis. *Infect. Immun.* 73, 5004–5014. doi:10.1128/IAI.73.8.5004-5014.2005.

Singh, P. P., Parra, M., Cadieux, N., and Brennan, M. J. (2008). A comparative study of host response to three Mycobacterium tuberculosis PE_PGRS proteins. *Microbiology* 154, 3469–3479. doi:10.1099/mic.0.2008/019968-0.

Singh, P., Rao, R. N., Reddy, J. R. C., Prasad, R., Kotturu, S. K., Ghosh, S., et al. (2016). PE11, a PE/PPE family protein of Mycobacterium tuberculosis is involved in cell wall remodeling and virulence. *Sci. Rep.* 6, 21624. doi:10.1038/srep21624.

Singh, S. K., Kumari, R., Singh, D. K., Tiwari, S., Singh, P. K., Sharma, S., et al. (2013). Putative roles of a proline–glutamic acid-rich protein (PE3) in intracellular survival and as a candidate for subunit vaccine against Mycobacterium tuberculosis. *Med. Microbiol. Immunol. (Berl.)* 202, 365–377. doi:10.1007/s00430-013-0299-9.

Singh, S. K., Tripathi, D. K., Singh, P. K., Sharma, S., and Srivastava, K. K. (2012). Protective and survival efficacies of Rv0160c protein in murine model of Mycobacterium tuberculosis. *Appl. Microbiol. Biotechnol.* 97, 5825–5837. doi:10.1007/s00253-012-4493-2.

Sultana, R., Vemula, M. H., Banerjee, S., and Guruprasad, L. (2013). The PE16 (Rv1430) of Mycobacterium tuberculosis Is an Esterase Belonging to Serine Hydrolase Superfamily of Proteins. *PLoS ONE* 8, e55320. doi:10.1371/journal.pone.0055320.

Thi, E. P., Hong, C. J. H., Sanghera, G., and Reiner, N. E. (2013). Identification of the *Mycobacterium tuberculosis* protein PE-PGRS62 as a novel effector that functions to block phagosome maturation and inhibit iNOS expression: PE-PGRS62 modulates macrophage function. *Cell. Microbiol.* 15, 795–808. doi:10.1111/cmi.12073.

Tiwari, B. M., Kannan, N., Vemu, L., and Raghunand, T. R. (2012). The Mycobacterium tuberculosis PE Proteins Rv0285 and Rv1386 Modulate Innate Immunity and Mediate Bacillary Survival in Macrophages. *PLoS ONE* 7, e51686. doi:10.1371/journal.pone.0051686.

Tiwari, B., Ramakrishnan, U. M., and Raghunand, T. R. (2015). The *M* *ycobacterium tuberculosis* protein pair PE9 (Rv1088)-PE10 (Rv1089) forms heterodimers and induces macrophage apoptosis through Toll-like receptor 4: The PE9-PE10 protein pair of *M. tb* is a TLR4 ligand. *Cell. Microbiol.* 17, 1653–1669. doi:10.1111/cmi.12462.

Tiwari, B., Soory, A., and Raghunand, T. R. (2014). An immunomodulatory role for the *Mycobacterium tuberculosis* region of difference 1 locus proteins PE35 (Rv3872) and PPE68 (Rv3873). *FEBS J.* 281, 1556–1570. doi:10.1111/febs.12723.

Tufariello, J. M., Chapman, J. R., Kerantzas, C. A., Wong, K.-W., Vilchèze, C., Jones, C. M., et al. (2016). Separable roles for *Mycobacterium tuberculosis* ESX-3 effectors in iron acquisition and virulence. *Proc. Natl. Acad. Sci.* 113, E348–E357. doi:10.1073/pnas.1523321113.

Tullius, M. V., Nava, S., and Horwitz, M. A. (2018). PPE37 Is Essential for *Mycobacterium tuberculosis* Heme-Iron Acquisition (HIA), and a Defective PPE37 in *Mycobacterium bovis* BCG Prevents HIA. *Infect. Immun.* 87, e00540-18, /iai/87/2/IAI.00540-18.atom. doi:10.1128/IAI.00540-18.

Tundup, S., Mohareer, K., and Hasnain, S. E. (2014). *Mycobacterium tuberculosis* PE25/PPE41 protein complex induces necrosis in macrophages: Role in virulence and disease reactivation? *FEBS Open Bio* 4, 822–828. doi:10.1016/j.fob.2014.09.001.

Xu, Y., Yang, E., Huang, Q., Ni, W., Kong, C., Liu, G., et al. (2015). PPE57 induces activation of macrophages and drives Th1-type immune responses through TLR2. *J. Mol. Med.* 93, 645–662. doi:10.1007/s00109-014-1243-1.

Yang, G., Luo, T., Sun, C., Yuan, J., Peng, X., Zhang, C., et al. (2017a). PPE27 in *Mycobacterium smegmatis* Enhances Mycobacterial Survival and Manipulates Cytokine Secretion in Mouse Macrophages. *J. Interferon Cytokine Res.* 37, 421–431. doi:10.1089/jir.2016.0126.

Yang, W., Deng, W., Zeng, J., Ren, S., Ali, M. K., Gu, Y., et al. (2017b). Mycobacterium tuberculosis PE_PGRS18 enhances the intracellular survival of M. smegmatis via altering host macrophage cytokine profiling and attenuating the cell apoptosis. *Apoptosis* 22, 502–509. doi:10.1007/s10495-016-1336-0.

Yu, Z., Zhang, C., Zhou, M., Li, Q., Li, H., Duan, W., et al. (2017). Mycobacterium tuberculosis PPE44 (Rv2770c) is involved in response to multiple stresses and promotes the macrophage expression of IL-12 p40 and IL-6 via the p38, ERK, and NF-κB signaling axis. *Int. Immunopharmacol.* 50, 319–329. doi:10.1016/j.intimp.2017.06.028.

Zumbo, A., Palucci, I., Cascioferro, A., Sali, M., Ventura, M., D’Alfonso, P., et al. (2013). Functional dissection of protein domains involved in the immunomodulatory properties of PE_PGRS33 of *Mycobacterium tuberculosis*. *Pathog. Dis.* 69, 232–239. doi:10.1111/2049-632X.12096.
